# The Volume and Capacity of Colonoscopy Procedures Performed at New York City Hospitals in 2002

**Published:** 2004-12-15

**Authors:** Jennifer C.F. Leng, Lorna E Thorpe, Gabe E Feldman, Pauline A Thomas, Thomas R Frieden

**Affiliations:** New York City Department of Health and Mental Hygieney; New York City Department of Health and Mental Hygiene, New York, NY; New York City Department of Health and Mental Hygiene, New York, NY; New York City Department of Health and Mental Hygiene, New York, NY; New York City Department of Health and Mental Hygiene, New York, NY

## Abstract

**Introduction:**

Colorectal cancer is the second leading cause of cancer death in New York City. In March 2003, the New York City Department of Health and Mental Hygiene recommended colonoscopy every 10 years as the preferred screening test for adults aged 50 years and older in New York City. To screen all eligible adults in New York City would require that approximately 200,000 colonoscopy exams be performed annually. As part of this recommendation, we evaluated current colonoscopy capacity in New York City hospitals.

**Methods:**

We surveyed endoscopy suite nursing or administrative staff at all 66 adult acute care hospitals performing colonoscopy in New York City. Data on colonoscopy procedures performed in 2002 were collected between February and June 2003.

**Results:**

All hospitals and two affiliated clinics responded. The number of hospital-based colonoscopy exams performed in 2002 was estimated to be 126,000. Of these, 53,600 (43%) were estimated to be for screening. Hospitals reported their maximum annual capacity to be 195,200, approximately 69,100 more than current practice. Reported barriers to performing more colonoscopy exams included inadequate suite time and space (31%), inadequate staffing (28%), and insufficient patient referrals (24%).

**Conclusion:**

In 2003, endoscopy suites at New York City hospitals performed approximately one quarter of the estimated citywide need of 200,000 screening colonoscopies. Procedures conducted in outpatient office settings were not assessed. Most endoscopy suites, particularly private hospitals, reported having the capacity to conduct additional procedures. Hospitals and endoscopy suites should prioritize the development of institutional measures to increase the number of persons receiving screening colonoscopy.

## Introduction

Colorectal cancer is the second leading cause of cancer deaths (after lung) in New York City (NYC) (1) and the leading cause of cancer death among nonsmokers (2). In 2000, 1600 New Yorkers died of colorectal cancer (1). In March 2003, based on findings from an advisory committee on colorectal cancer screening (Citywide Colon Cancer Control Coalition), the New York City Department of Health and Mental Hygiene (NYC DOHMH) recommended colonoscopy every 10 years as the preferred colorectal cancer screening test for average-risk asymptomatic men and women aged 50 years and older in NYC (2,3). Colonoscopy is highly sensitive, examines the entire colon, and allows for screening, diagnosis, and polypectomy in a single visit. While colonoscopy is one of a series of recommended options in all major national colorectal cancer screening guidelines (4-7), few institutions have endorsed it as the preferred screening option. Findings from the National Polyp Study, however, suggest that periodic colonoscopy could prevent 76% to 90% of colon cancers (8). The NYC DOHMH recommendation was based on this estimated effectiveness of colonoscopy in addition to the desire to reduce patient and provider confusion about multiple screening options.

Nationally, concern over increased colonoscopy demand and insufficient capacity has raised the question of whether performing colonoscopies on all eligible adults aged 50 and older is feasible (9). In some U.S. cities, physicians cite waiting lists of up to eight months, and in extreme situations, waiting lists have been closed (9). New York City has a high concentration of specialists and teaching hospitals and therefore may have a greater capacity to perform colonoscopies compared with other communities (10). The NYC DOHMH sought to evaluate current colonoscopy volume and reported maximum capacity in all NYC acute care hospitals.

## Methods

### Study population

We conducted a telephone survey of nursing or administrative staff at all NYC adult acute care hospitals from February to June 2003 (11). This included 55 voluntary hospitals, three Veterans Administration (VA) hospitals, and 11 public hospitals (which provide care to New Yorkers regardless of their ability to pay). NYC DOHMH staff telephoned hospital endoscopy suites and interviewed the nurse manager or his or her designee. NYC DOHMH staff requested to interview the clinic or nurse manager or the person most able to accurately provide data on the number of colonoscopy exams performed at that endoscopy suite. If key staff were unavailable at the time of the call, a follow-up telephone call was arranged.

At the close of the interview, NYC DOHMH staff also asked if there were other clinical sites within the same facility (or affiliated with the facility) that performed colonoscopy exams. If so, NYC DOHMH staff interviewed staff at these additional sites. Every distinct clinical site that performed colonoscopy procedures and was officially affiliated with the originally targeted facility was included in the survey. Specific departments, such as Gastroenterology and General Surgery, were not separately contacted.

### Survey

Using structured survey questions, interviewers asked respondents to identify the type of clinic in which colonoscopy exams are performed, the number and specialty of physicians who perform the procedure, and the waiting time to schedule a patient. Respondents were asked the total number of exams performed during the past year (2002), and the approximate percentage (<25%, 25%–50%, 50%–75%, or >75%) performed for screening purposes. They were then asked to provide the maximum number of exams that *could* be performed per month. Final questions probed respondents about barriers to performing at full capacity and willingness to receive more colonoscopy referrals. No patient-level information was collected.

### Statistical analysis

The citywide need for screening colonoscopy exams was estimated assuming a steady-state population structure. We divided the 2000 census count of New Yorkers aged 50 and older (2,102,578) by the recommended screening colonoscopy interval (10 years) (1). We estimated that to screen all 2 million eligible New Yorkers every 10 years, NYC would need to perform approximately 200,000 colonoscopy exams per year.

Survey variables were analyzed using descriptive statistics. Each hospital reported the total number of exams performed in 2002; the sum of these numbers is the estimated total number of exams performed in hospitals citywide. The estimated number of exams performed for screening purposes in 2002 was calculated by multiplying the midpoint of a given clinic's reported screening percentage category by the total number of reported colonoscopy exams performed in 2002 for that clinic. The same calculation was performed using the low and high endpoints of a given screening percentage category to give an estimated range of the number of exams performed for screening for each clinic. The estimated maximum number of colonoscopy exams an endoscopy suite could perform per year was determined by multiplying the reported maximum number of exams feasible per month by 12.

Potential annual residual capacity was estimated by subtracting the number of exams performed in 2002 from the estimated maximum annual number of exams that could be performed. All estimated values were rounded to the nearest hundred because of the measurement tool's lack of precision. The estimated annual maximum number of exams performed and the estimated annual potential residual capacity were calculated using the exact numbers reported and then rounded to the nearest hundred. Descriptive results were presented according to type of hospital (voluntary/VA vs public). Data were analyzed using SAS, version 8 (SAS Institute, Inc, Cary, NC).

## Results

We contacted staff at all 69 acute care hospitals in NYC. Three hospitals did not have endoscopy suites and were excluded from the survey. Two hospitals had additional affiliated endoscopy suites; staff from these sites were included in the survey for a total of 68 sites (Table 1). Most hospitals were voluntary/VA hospitals (84%); the rest (16%) were public hospitals.

The majority of staff interviewed (51%) were nurses in supervisory positions, including nurse managers, charge nurses, nurse directors, and nurse supervisors. Eighteen percent were managers or executive administrators, and 10% were physicians or medical directors. The remaining respondents (21%) were medical and administrative ancillary staff responsible for maintaining endoscopy suite colonoscopy schedules. Respondents reported that a total of 963 physicians were performing colonoscopy exams in these endoscopy suites at the time of the survey. Ninety-three percent of physicians were gastroenterologists, and the remaining 7% were colorectal or general surgeons. The median number of physicians performing colonoscopy exams at public endoscopy suites was five, compared with 12 at voluntary/VA endoscopy suites. The median waiting period to schedule a routine screening colonoscopy was 49 days (range, 21–150 days) at public endoscopy suites and seven days (range, 0–90 days) at voluntary/VA endoscopy suites.

The total number of reported colonoscopy exams performed in acute care hospitals in NYC in 2002 was estimated to be 126,000 (Table 2). Endoscopy suites at voluntary/VA hospitals performed an estimated 117,200 (93%) of the total estimated colonoscopy exams; public hospitals performed 8800 (7%). The total number of exams performed for screening was estimated to be 53,600 (range, 37,800–69,000). Sixty-one endoscopy suites (90%) were able to provide data on both the number of exams performed in 2002 and the estimated maximum annual number of exams that could be performed; of these, 89% reported a maximum capacity that was higher than the volume reported for 2002. If maximum capacity as reported by suites was achieved, approximately 69,100 additional colonoscopy exams could be conducted annually (potential residual capacity). Most of the potential residual capacity (88%) was reported by voluntary/VA endoscopy suites; 12% was reported by public endoscopy suites.

All public hospitals and 50 of 57 voluntary/VA hospitals reported at least one barrier to performing more colonoscopy exams. The most commonly reported barriers were inadequate suite time and space (31%), low physician-staffing levels (28%), low nurse- and/or technician-staffing levels (28%), and insufficient patient referrals (24%). Respondents at public facilities more frequently cited staffing shortages compared with private facilities (45% [public] vs 25% [private] needed more physicians; 64% [public] vs 21% [private] needed more nurses/technicians). At public facilities, respondents also more often described patient no-shows and cancellations (27% [public] vs 4% [private]) as barriers to performing more exams and less frequently described a lack of referrals as a barrier (9% [public] vs 26% [private]). Eighty-eight percent of suites responded yes to the question "Would you like more referrals?" and 71% responded yes to "Would you like to be listed for referrals?" Sixty-three percent of suites reported that they were willing to submit a monthly colonoscopy report to the health department documenting the number of exams performed.

## Discussion

We found that approximately 126,000 colonoscopies are being conducted annually in NYC hospitals, almost half (43%) of which are performed for screening purposes. Hospitals reported a potential to conduct an additional 69,100 procedures if barriers could be sufficiently addressed. In 2002, hospital-based endoscopy suites performed approximately one quarter of the estimated annual need of 200,000 screening exams. Effective hospital-based improvements could potentially double this number by enabling endoscopy suites to perform closer to maximum capacity. The actual total volume of colonoscopy procedures being conducted in NYC is unknown, because we currently lack data on the volume of procedures performed in outpatient office settings.

Public hospital endoscopy suites represented 16% of all suites surveyed, yet they conducted only 7% of reported procedures, suggesting a lower overall volume than in the private sector. Nonetheless, public hospitals also reported the capacity to increase the number of procedures they performed (12% of total additional capacity). Although this suggests that public facilities, not just private facilities, may be functioning below full capacity, the long waiting period to schedule a screening colonoscopy, the low number of exams performed (relative to private facilities), the more severe physician staffing shortages, and the more frequent patient cancellations all imply that public hospitals face more obstacles when striving to operate at maximum capacity.

Findings from this survey suggest that, if barriers were adequately addressed, NYC would have sufficient screening capacity in hospital endoscopy clinics to meet much of the demand generated by a focused colonoscopy campaign. Additional capacity is currently concentrated in private facilities, which primarily serve patient populations with health insurance. According to a population-based survey conducted in 2002, only about half of New Yorkers over the age of 50 reported ever having had a colonoscopy or sigmoidoscopy, leaving nearly 1 million adults at greater risk for undetected colon cancer (12). Hospitals and clinics should develop institutional measures to increase the number of persons receiving screening colonoscopy. Regular reminders to primary care physicians to refer patients for colonoscopy, rapid referral systems to expedite the referral process, protocols to bypass the initial visit with the endoscopist, and greater efficiency in colonoscopy procedures could increase the number of colonoscopy exams performed. Community-based organizations, advocacy groups, local government, and the medical community could advocate for legislative changes to increase reimbursement, reduce copays, and mandate insurance coverage for screening colonoscopy exams. These same groups should also work to increase public awareness and further educate providers about colorectal cancer screening. As a result of this study, the NYC DOHMH developed a colonoscopy surveillance system to track the volume of colonoscopy procedures performed in NYC hospitals; this system will allow the NYC DOHMH to monitor the impact of citywide efforts to increase screening colonoscopy rates.

Particular consideration should be given to increasing the number of colonoscopy exams performed on uninsured and low-income patients, who often face significant barriers to health care. Facilities may be able to improve the efficiency of endoscopy suites and decrease patient cancellations by the use of patient navigators (staff members designated to help patients negotiate complex public hospital systems). Shifting some of the need of the uninsured, low-income population to private hospitals may also provide a partial solution and could be accomplished through partnerships among local hospitals.

This study has limitations. We did not attempt to provide data on the complete universe of colonoscopy procedures in NYC, because information on the number and location of outpatient office settings where colonoscopy procedures are performed was unavailable. However, in NYC, colonoscopy procedures performed in hospital endoscopy suites do likely represent a significant proportion of all colonoscopy procedures. One study estimated that 25% of the estimated 35 million outpatient procedures performed nationwide in 2001 were performed in physicians' offices (13). In NYC, this proportion may be similar or even lower, due to the relatively high proportion of uninsured and Medicaid patients in NYC (approximately 19% of persons aged 50 and older in 2002) (14) and the high concentration of hospitals in NYC. Because of low reimbursement, colonoscopy procedures for Medicaid patients are generally only performed at hospital endoscopy suites by hospital-employed salaried physicians (15).

Another limitation was that our estimated citywide need for 200,000 annual screening colonoscopy exams was based on the 2000 census count and assumed a steady-state population structure. This was likely an overestimate, as we did not account for those who have medical contraindications to colonoscopy, those who have already been screened, and those who will absolutely refuse the procedure. This overestimate may be slightly offset by high-risk persons who require more frequent colonoscopy for surveillance; additionally, as more eligible persons undergo screening, more colonoscopy procedures will be needed to perform surveillance on those in whom polyps were identified.

Finally, respondents may have overestimated the annual maximum number of exams that could be performed. Respondents were not asked specifically to report whether they were considering any additional resource investment, such as staff or equipment, in estimating the maximum number of exams that could be performed and therefore may have based their estimate on endoscopy suite availability only.

Our study demonstrates a feasible method for obtaining data on colonoscopy volume and capacity at hospitals in an urban area, either as a one-time study or as a study repeated at regular intervals. In June 2003, the NYC DOHMH began collecting similar data on colonoscopy procedures on a quarterly basis. These data are used to assess the impact of ongoing agency efforts to increase the proportion of eligible persons who undergo screening colonoscopy exams.

In 2003, the NYC DOHMH created a coalition of key individuals and organizations that share an interest in decreasing the incidence and mortality of colon cancer, the Citywide Colon Cancer Control Coalition (C5). The mission of the C5 is to improve citywide colon cancer prevention and control by increasing awareness and screening. As part of these efforts, the C5/NYC DOHMH developed guidelines that state: "Most people 50 years of age and older should undergo colonoscopy every 10 years. Annual fecal occult blood testing (FOBT) is an acceptable, although not optimal, alternative for those unwilling or unable to undergo colonoscopy. Persons at high risk for colorectal cancer should begin screening with colonoscopy at age 40 or earlier" (2,3). Removing institutional barriers to increasing screening capacity, as well as improving access to care, are critical tasks for C5 coalition members to address; a concerted citywide effort is essential to achieving these goals.

## Figures and Tables

**Table 1:** Characteristics of Surveyed Endoscopy Suites in New York City Hospitals, 2003

	**No. (%)**
**Hospitals contacted^a^**	69
**Hospitals performing colonoscopy**	66
**Additional affiliated clinics**	2
**Endoscopy suites surveyed**	68 (100)
**Voluntary/VA^b^**	57 (84)
**Type of endoscopy suite**	
Inpatient and outpatient^c^	55
Outpatient, free-standing^d^	2
**Public**	11 (16)
**Type of endoscopy suite**	
Inpatient and outpatient	11

a One hospital was a hospital center consisting of two separate hospital units; this was analyzed as one hospital.

b VA = Veterans Administration.

c One hospital used both an inpatient/outpatient suite and an operating room to perform colonoscopy exams.

d One outpatient, free-standing endoscopy suite was actually an outpatient office practice affiliated with a hospital.

**Table 2:** Reported and Estimated Volume of Colonoscopy Exams Performed at New York City Hospitals, 2002, and Estimated Potential Capacity

	**All endoscopy suites N = 68 **	**Voluntary/VA^a^ N = 57 **	**Public N = 11**

**Reported number of exams performed, 2002^b^**			

Median (range)	1290 (260-7000)	1507 (260-7000)	821 (400-1450)
Estimated total	126,000	117,200	8800

**Estimated number of exams performed for screening, 2002^c^**			

Midpoint (range)	53,600 (37,800-69,000)	49,800 (35,200-64,200)	3800 (2700-4800)

**Reported maximum number of exams per month^d^**			

Median (range)	200 (30-800)	200 (30-800)	150 (45-320)
Estimated total	16,300	14,800	1400
**Estimated annual maximum number of exams^e^**	195,200	177,800	17,400
**Estimated annual potential residual capacity^f^**	69,100	60,600	8500

a VA = Veterans Administration.

b Two voluntary/VA and one public endoscopy suite responded “don't know” to this question.

c Data missing from two voluntary/VA and one public endoscopy suite.

d Four voluntary/VA and two public endoscopy suites responded “don't know” to this question.

e Values were rounded to the nearest hundred; numbers do not add up to the “Reported maximum number of exams per month" x 12.

f Values were rounded to the nearest hundred; numbers do not add up to the “Reported number of exams performed in 2002” subtracted from “Estimated annual maximum number of exams.”
